# Consumer Engagement in the Design of Educational Nutrition Information for Older Adults and Their Caregivers: A Scoping Review

**DOI:** 10.1016/j.advnut.2025.100401

**Published:** 2025-03-06

**Authors:** Adeline Lau, Adrienne M Young, Chad Han, Elizabeth M Miller, Mia EL Heim, Michelle D Miller

**Affiliations:** 1 Caring Futures Institute, College of Nursing and Health Sciences, Flinders University, Bedford Park, South Australia, SA, Australia; 2 Australian Frailty Network, the University of Queensland, Brisbane, QLD, Australia; 3 Centre for Health Services Research, the University of Queensland, Brisbane, QLD, Australia; 4 Nutrition Research Collaborative, Royal Brisbane and Women’s Hospital, Brisbane, QLD, Australia; 5 Consumer Partnering Unit, Metro South Hospital and Health Service, Brisbane, QLD, Australia

**Keywords:** codesign, patient-centered care, user-centered design, consumer participation, community-based participatory research, health information

## Abstract

Consumer engagement is important to design high-quality educational nutrition information that holistically addresses consumers’ needs. This can occur through consultation and feedback mechanisms like surveys or focus groups, consumer expert panels or advisory boards to provide the consumers’ perspective, or through participatory research methods. The extent of consumer engagement also varies with differing levels of influence over the decision-making process. This systematic scoping review aimed to explore and synthesize the extent to which consumers are engaged in designing various types of educational nutrition interventions, the methods and levels of consumer engagement, and its impact on the resulting educational nutrition information presented. We comprehensively searched Medline via OVID, Scopus, Web of Science, CINAHL, and PsycINFO. Each article was independently screened by 2 authors by title and abstract. Two reviewers independently assessed the full text of the remaining articles for eligibility. Two authors independently extracted data from the 36 final articles (15 original and 21 substudies), including consumer engagement assessment per the International Association of Public Participation (IAP2) spectrum. Fourteen of the 15 studies obtained input from consumers to inform the design of the educational nutrition information in terms of content, design, wording, and platform. However, consumer engagement across the studies mostly sat within the “Consult” and “Involve” level of the IAP2 spectrum, with only 1 study achieving a “Collaborate” engagement level. This suggests a low level of genuine consumer partnership in the studies to date. Consumer engagement across the studies differed on how and the extent to which consumers were engaged in designing educational nutrition information. Greater emphasis on shared decision-making and collaborating with consumers right from the start is key to ensuring that educational nutrition information designed for them best addresses their needs and preferences, which potentially translates to better health outcomes.

**Statement of significance**

This scoping review has synthesized and mapped the types and level of consumer engagement in the design of educational nutrition information targeted toward older adults and their caregivers. The impact of consumer engagement has also been explored, although further analyses are needed to quantify the impact and effectiveness of consumer engagement on health and research outcomes.

## Introduction

### Background

Nutrition education is a core component of dietetic practice and health promotion, which has a significant impact on preventing and managing chronic diseases [1,2]. Yet, nutrition education to influence changes to an individual’s diet is more complex than simply handing out an educational nutrition brochure or handout. Although these written materials can increase one’s nutrition awareness and knowledge, generally, it needs to be complemented with other nutrition education strategies to allow for effective improvements in nutrition behaviors and attitudes [3,4]. Recognizing the intention-behavior gap [5] where improved nutrition knowledge with an intention to improve one’s diet does not necessarily always translate to positive dietary behavior changes is important, so that key actions are taken to address and bridge that gap. Moreover, as individuals attempt to enact their intentions (e.g. to improve their protein intake), they may face self-regulatory problems, such as problems with getting started due to indecision (e.g. uncertainty of how to increase protein intake due to lack of knowledge of dietary protein sources) or a lack of preparation to perform the desired behavior (e.g. did not purchase dietary protein sources for consumption) [5]. Therefore, well-designed educational nutrition information that incorporates behavior change techniques and personalized feedback tailored to address the specific problems, needs and concerns of each target individual is essential in bridging this intention-behavior gap. In addition, the nutrition education should be presented in a way that best caters to the intended audiences’ learning preferences [3].

Older adults have unique learning needs. A decline in information processing with age [6] requires nutrition educational resources for older adults to be designed in a way that accommodates their specific learning preferences. To ensure that older adults’ unique needs and preferences are holistically addressed, their input into the planning and design of the information is important. Input from the intended audience ensures that the educational nutrition information presented is easy to comprehend in their preferred format, and more importantly, is well-tailored to address their main concerns, which is a key to improving their diet and health outcomes [7]. A review by Goodman and Lambert [8] describes the preferences of older adults for patient education materials in terms of layout, design, and content, highlighting older adults preferences for larger font size, use of color and informative headings, as well as the use of visual aids [8]. Current nutrition educational information resources can also be improved in terms of actionability, where nutrition advice is broken down into simple steps and instructions are provided on how to utilize the information provided [9].

Furthermore, sociocultural factors, in addition to personal food beliefs and perceptions, can influence dietary behavior in older adults from diverse backgrounds, and should be considered [10]. The systematic mapping review by Osei-Kwasi et al. [10] revealed that sociocultural factors such as cultural identity, and the wish to preserve one’s traditional food identity, along with religious beliefs, and beliefs pertaining to traditional foods, all have the ability to influence one’s dietary behavior and eating habits. These findings were supported by Asamane et al. [11], who found that personal, social, cultural, and environmental factors were the key factors influencing eating behaviors in community-dwelling ethnically diverse older adults. Therefore, practicing good cultural competence when designing educational nutrition information for older adults and their caregivers, especially for those from diverse backgrounds, is important and can enhance the effectiveness of the nutritional intervention in promoting positive dietary behavior changes.

Apart from good cultural awareness, involving end-users in the decision-making process when designing educational nutrition information is paramount to ensure their specific needs and concerns have been considered. End-user participation in the decision-making process at both the individual level around their own health, treatments and illness-management, and more broadly at a health service level around health policy development, service design and delivery [12], as well as in healthcare planning, monitoring and evaluation [13] has been termed “consumer engagement.” In the context of healthcare, consumers include individual patients, their caregivers, family and friends, as well as consumer representatives advocating for the interests of patients, caregivers or specific client groups [14]. Effective consumer engagement requires health services to work alongside consumers, family, and carers as equal partners in their own care, which is essential for quality health service and patient-centered care [15]. As such, consumer engagement in healthcare interventions and research has become recognized as increasingly important, although the types, extent and level of consumer engagement can vary widely. Furthermore, the term “consumer engagement” is also often used interchangeably with other similar terms, such as “public and patient involvement” [16] and “citizen engagement” [17], all of which describes meaningful involvement of individuals in various planning, decision-making and evaluation processes [17].

Consumer engagement can enhance the quality and direction of research and healthcare interventions. Some potential benefits of consumer engagement in healthcare include generating research, practice, and consumer information that is more sensitive to the needs and concerns of consumers [18], which will translate to enhanced receptiveness to research or practice interventions and recommendations. As consumers are end-users of the healthcare system and research outcomes, engaging consumers in healthcare and research not only provides valuable insight into consumers’ unique or complementary perspectives compared with those of healthcare professionals and researchers [18], but it also provides consumers with a sense of ownership and empowerment over their own health, which potentially results in better health outcomes. As consumers are experts and “the voice” in their own health, their input adds evidence-based value and genuine insights into what consumers think and feel, rather than relying on second-hand, hearsay-assumed experiences. In addition, practicing cultural competence by recognizing the diversity within communities and individuals in those communities, all with their own beliefs and values, is vital for effective consumer engagement [19]. This ensures that healthcare interventions and research outcomes are culturally relevant and can meaningfully meet the specific needs of the intended audience. Furthermore, consumer engagement promotes transparency and accountability, which enhances the quality of the healthcare intervention or research. Thus, it is undeniable that consumers’ input in healthcare and research is beneficial.

Consumer engagement may take various forms, such as through consultation and feedback processes like surveys or focus groups, consumer expert panels or advisory boards to provide their consumers’ perspective. Consumers can also be engaged through participatory research methods where they provide input at different stages of the research intervention. Consumer engagement can occur at the initial planning stage, where consumer views are sought via a needs assessment to determine what consumers’ needs and preferences are. Consumers can also provide input at the design and production stages to inform the content, design, and platform of the educational nutrition information to be presented. Furthermore, consumer engagement can occur at the implementation and evaluation stages, where educational nutrition interventions or prototypes are implemented and evaluated for their feasibility, acceptability, usability, and effectiveness.

Additionally, the extent of consumer engagement may also vary as there are many ways that consumers can be engaged, with differing levels of influence and power over the decision-making process and outcomes. One way to describe this is by using the International Association for Public Participation (IAP2) Public Participation Spectrum [20]. From one end of the public participation spectrum of “Inform” to the other end of the spectrum to “Empower,” there is increasing impact and influence from consumers as decision-makers [20].

### Rationale and objective/s

Although previous reviews have provided insight into codesign techniques used in nutrition research [21] and consumer codesign in nutrition interventions [22], further insight on consumer engagement in research involving educational nutrition information specifically targeted for older adults is needed. Given the unique needs and preferences of older adults, it is important that information targeted for this population group is tailored to their needs, and presented and delivered in a meaningful, acceptable, and efficient way. Although Meloncelli et al. [22] reviewed the outcomes of consumer codesign in their scoping review, studies that engaged consumers at the “Inform,” “Consult,” or “Involve” IAP2 levels were excluded. Thus, further exploration into consumer engagement in the development and design of educational nutrition information, and on the resulting “end-product” for the varying levels of consumer engagement across the entire IAP2 spectrum [20] is warranted. From nutrition information being presented in a more traditional paper-based form, such as in educational brochures or leaflets, to more current digital platforms, such as mobile applications or websites, the ways in which consumers are engaged across these different platforms are also likely to be different. Therefore, with a focus on older adults, this systematic scoping review aims to explore and synthesize the extent to which consumers are engaged in designing various types of educational nutrition interventions, the methods and levels of consumer engagement, and the impact of consumer engagement on the resulting educational nutrition information presented.

## Methods

### Protocol and registration

This scoping review was conducted using a systematic approach guided by the Joanna Briggs Institute methodology for scoping reviews [23] and is reported in line with the PRISMA extension for Scoping Reviews Checklist [24] (Supplemental Table 1). A preliminary search was conducted on 9 May, 2023 for previous scoping reviews on the topic in Medline via OVID. The protocol for this scoping review has been registered in the Open Science Framework (OSF) Registry [25] on 13 September, 2023 and updated on 27 September, 2023 to provide better precision to the terms and methods used in our protocol (https://osf.io/28wtq, assessed on 27 September, 2023). Any deviations and updates made to our registered OSF protocol can be found in Supplemental Table 2.

### Eligibility criteria

The eligibility criteria for this review is outlined next, according to the Participants, Concept and Context (PCC) structure [23]:

#### Participants

In this review, eligible studies must have included consumers, who were defined in the context of healthcare to include: patients, unpaid carers, informal caregivers, users of health services, and members of the public who are potential recipients of healthcare or health programs [18]. Consumers also include families of patients and people who have lived experience of a health issue [26], and consumer representatives advocating for the interests of patients, caregivers or specific client groups [14]. In addition, only studies that have reported a mean or median age or ≥50% of consumers and/or target audience involved ≥65 y of age were included.

#### Concept

Studies that included these 2 concepts were included: *1*) consumer engagement in the design of *2*) educational nutrition information for older adults. Studies that included any IAP2 level [20] of consumer engagement (Table 1) [20], at any stage of the design and/or development of the educational nutrition information across any medium or platform (e.g. mobile applications, websites, handouts, etc.) were eligible. It is also important to note that the term consumer “engagement” can be confusing as it is often used interchangeably with other related terms such as “participation,” “involvement,” and “collaboration” [26]. For this review, the term consumer "engagement" will be used to describe consumer input across the studies, to minimize confusion with the terminologies used in the International Association of Public Participation (IAP2) spectrum [20] that will be used to describe the level of consumer engagement in each of the included studies later in this review.

**TABLE 1:** Consumers’ impact on the decision-making process per the IAP2 Public Participation Spectrum.

IAP2 level [20]	Promise to the public [20].	Consumers’ impact on the decision-making process.
Inform	We will keep you informed.	Consumers are not part of the decision-making process but rather will be kept informed to assist them in understanding the problem or solutions available.
Consult	We will keep you informed, listen to and acknowledge concerns and aspirations, and provide feedback on how public input influenced the decision.	Consumers’ input may influence the decision-making process.
Involve	We will work with you to ensure that your concerns and aspirations are directly reflected in the alternatives developed and provide feedback on how public input influenced the decision.	Consumers are recognized as one of the decision-makers.
Collaborate	We will look to you for advice and innovation in formulating solutions and incorporate your advice and recommendations into the decisions to the maximum extent possible.	Consumers have an equal say in the decision-making process.
Empower	We will implement what you decide.	Consumers have the final say in terms of decision-making.

Abbreviation: IAP2, International Association for Public Participation.

We excluded the following:

• Studies where consumers were involved but nutrition was not a component of the end-product or intervention.
• Studies where consumers were involved but no "end-product" or educational nutrition information was developed or presented (e.g. focus group sessions were conducted with consumers to explore their preferences for specific educational nutrition information and features, but no resulting educational nutrition information or “end-product” was designed or presented based on the focus group findings [27]).
• Studies relating to nutrition education curriculum for academic purposes.
• Studies where the consumers’ or target audiences’ mean or median age were not reported.

#### Context

Included studies were peer-reviewed, primary, quantitative, qualitative, and mixed-method studies from any country. No language or date restrictions were imposed. Review articles and conference abstracts were excluded.

### Information sources, search and selection of sources of evidence

The search strategy was developed by the author AL and revised by 2 authors (AMY and MDM). In addition, the keywords and controlled vocabulary terms in the search strategy were reviewed by a research librarian. A search was conducted on 15 May, 2023 across these 5 electronic databases: Medline via OVID, CINAHL, Scopus, Web of Science, and PsycINFO. The respective search strategy used in each of these databases can be found in Supplemental Table 3. To ensure a comprehensive map of the literature is provided, no restrictions were placed on the publication dates of sources or the language in which it is published. The sources obtained from each database search were imported into the Covidence software [28] which subsequently removed any duplicates. Three authors (AL, CH, and MH) independently conducted the screening and selection of articles via Covidence [28], with each source checked independently by 2 authors at the title, abstract and full-text stages. Any discrepancies regarding the inclusion or exclusion of articles were resolved via consensus among the 3 authors.

### Data charting process, data items and synthesis of results

Data extraction was performed independently by 2 authors (AL and CH) using a data extraction form. This form was reviewed by AMY and MDM before it was piloted by AL before use, to ensure that all relevant data were extracted. The 2 authors (AL and CH) then reviewed their independent data extraction forms together until a consensus was reached on the data extracted. Information extracted from the relevant articles included the types of educational nutrition interventions consumers were involved in, the main topic of focus of each intervention, the types of consumers involved, and the level and impact of consumer engagement across IAP2 spectrum. In addition, a third author (AMY) experienced in consumer engagement and the IAP2 spectrum reviewed all articles to assess the level of consumer engagement across the included studies. The IAP2 Public Participation Spectrum ranging from "Inform" through "Empower" was used to assess each study’s level of consumer engagement, which was dependent on how much impact consumers had in terms of the decision-making process [20]. Our consumer advisor also assessed each study’s level of consumer involvement from a consumers’ perspective, and upon discussions with the lead author (AL), an agreement on the IAP2 classifications for each study was reached. Findings on the outcomes of the included studies in this review were not analyzed as this review aim to map and summarize the extent to which consumers are engaged rather than the effectiveness of their engagement or of the nutrition education, in line with the purpose of a scoping review [29].

### Consumer engagement in scoping review

In a genuine attempt to include the perspective of consumers in synthesizing the information obtained from the included studies, an experienced consumer (LM: current active user of the healthcare system and consumer advisor) was included as part of this scoping review team and is one of the authors of this review. Her involvement included working closely alongside the lead author (AL) through 3 online 1-h meetings to provide input on this review from a consumer’s perspective. This involved an initial meeting to discuss her role, what and how she would like to contribute as a consumer, and if there were any areas in which the research team could further support her in her role as a consumer advisor on this review. In addition to providing IAP2 assessments for each study, our consumer advisor also provided input and feedback on the level of consumer engagement across the studies, as well as how consumer engagement can be improved. In addition to remuneration for her time and contributions (in line with rates set by Health Consumers Queensland, AU $45/h), authorship on this article further acknowledged her valuable input. The Guidance for Reporting Involvement of Patients and the Public-Short Form (GRIPP2-SF) [30] has been used to ensure quality and consistent reporting of the consumer’s engagement in this scoping review (Supplemental Table 4).

## Results

### Selection of sources of evidence + PRISMA diagram

**FIGURE 1:**
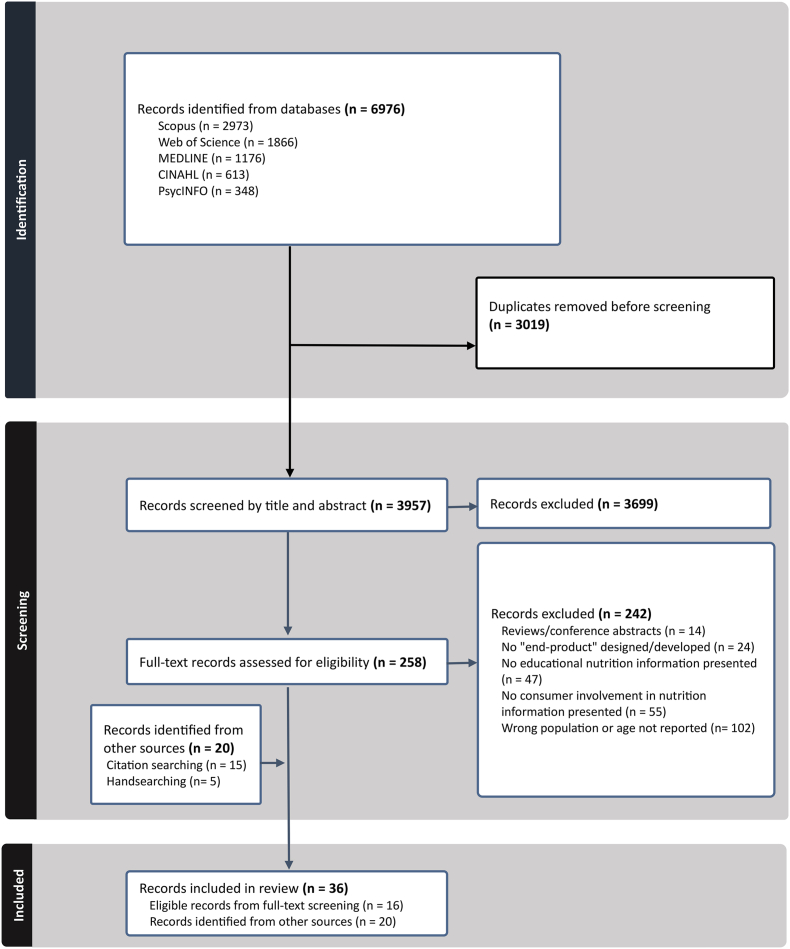
PRISMA 2020 flow diagram.

A total of 6976 records were obtained from the initial search across the 5 electronic databases with a resulting 3957 records after the removal of duplicates (Figure 1). After the title and abstract screening, 258 full-text records remained to be assessed for eligibility with a total of 16 records meeting the eligibility criteria and were included in this scoping review for the synthesis of results, along with 20 other records that were identified through either citation searching (*n* = 15) or handsearching (*n* = 5). As such, a total of 36 articles have been identified that report on 15 original studies. It is to be noted that although 4 studies identified through citation searching or handsearching did not formally meet the inclusion criteria for age [[31], [32], [33], [34]] (i.e. mean or median age of consumers or target audience was <65 y or >50% were <65 y), they met all the other eligibility criteria and were included within this review as they provided additional information about consumer engagement relevant to the formative work [31,33,34] or evaluative work [32] for each of their respective original studies.

### Study characteristics

The articles included in this review had publication dates ranging from 2008 to 2023. All but 2 studies [35,36] (*n* = 13) involved multiple phases with various study designs used across each of these phases in the development of the educational nutrition information. The platform used to deliver the educational nutrition information varied from websites [35,37,38] (*n* = 3), to paper-based [39,40] (*n* = 2), to tablet [41] (*n* = 1), or mobile application based [36] (*n* = 1), with a majority of studies incorporating multicomponent interventions using a mix of various platforms [[42], [43], [44], [45], [46], [47], [48], [49]] (*n* = 8). Some of the studies presented nutrition information focusing on chronic diseases, of which 1 study looked at overall self-management of chronic diseases [42], whereas others focused on cardiovascular disease [37,49] (*n* = 2), type 2 diabetes [35] (*n* = 1), chronic kidney disease (CKD) [38] (*n* = 1), and prostate cancer [44] (*n* = 1). One study presented nutrition information on perioperative care postcardiac surgery [36] (*n* = 1), and the rest of the studies were targeted more toward older adults, with topics focused on pressure ulcer prevention [43,45] (*n* = 2), malnutrition [46,48] (*n* = 2), eating advice for denture wearers [39] (*n* = 1), food-related activities in dementia [40] (*n* = 1), Mediterranean diet for older adults [47] (*n* = 1), and nutrition and physical activity for older adults in rehabilitation [41] (*n* = 1). An overview of the study characteristics can be found in Table 2 [[31], [32], [33], [34], [35], [36], [37], [38], [39], [40], [41], [42],[44], [45], [46], [47], [48], [49], [50], [51], [52], [53], [54], [55], [56], [57], [58], [59], [60], [61], [62], [63], [64], [65], [66]].

**TABLE 2:** Overview of study characteristics.

Original study/substudies	Country	Type of nutrition information presented	Focus of nutrition information
Al-Sultani et al. [39] (2023)	United Kingdom	Educational leaflet	Eating advice for complete denture wearers
Chaboyer et al. [43,50,51] (2016)	Australia	Multicomponent: Care bundle intervention (INTACT: INTroducing A Care bundle To prevent pressure injury) consisting of a 5-min DVD, information brochure and poster on pressure ulcer prevention	Pressure ulcer prevention
Chudyk et al. [36] (2021)	Canada	Mobile application	Cardiac surgery recovery
Coales et al. [35] (2023)	United Kingdom	Online Guided Self-Help (GSH) intervention: an adapted online version of the Working to Overcome Eating Difficulties GSH intervention	Management of binge eating in adults with type 2 diabetes
Donald et al. [31,33,34,38,52,53,54] (2021)	Canada	Web-based self-management support	Chronic kidney disease
Happe et al. [41,55] (2022)	Germany	Tablet-based e-Coach	Nutrition and physical activity for older adults in rehabilitation
Ilie et al. [44,56,57] (2023)	Canada	Multicomponent: Online home-based Prostate Cancer Patient Empowerment Program (PC-PEP) consisting of daily e-mails with video instructions providing education, patient activation, and empowerment on healthy living including physical and mental health, dietary recommendations, social support, physical and pelvic floor fitness, stress reduction using a biofeedback device, social connection and intimacy, and social support	Physical, mental, and social support intervention to prevent psychological distress among men undergoing curative prostate cancer treatment
Jongstra et al. [37,58, 59] (2017)	The Netherlands, Finland, and France	Web-based interactive platform	Cardiovascular disease risk
Papachristou et al. [40,60] (2018)	United Kingdom	Educational booklet	Food-related activities (shopping, preparation and eating) in dementia
Payne et al. [46,[61], [62], [63]] (2021)	United Kingdom	Multicomponent: “Eat well, feel well, stay well” Intervention consisting of a series of booklets, a food list and goal cards for patients	Malnutrition
Price et al. [32,49] (2009)	United Kingdom	Multicomponent: Brief lifestyle advice and UKPDS (UK Prospective Diabetes Study) Risk Engine	Cardiovascular disease risk
Roberts et al. [45] (2016)	Australia	Multicomponent: Patient-centered nutrition intervention consisting of a simple educational brochure on nutrition for pressure ulcer prevention (PUP), a standard food chart for patients to record their intake and nutritional goal setting guided by the researcher	Pressure ulcer prevention
Terp et al. [48,[64], [65], [66]] (2022)	Denmark	Multicomponent: Educative Nutrition Intervention (ENI) consisting of a tablet-based Food'n'Go program and interdisciplinary facilitation of patient participation including a poster and pamphlet with key messages of the ENI and a user guide for Food’n’Go for patients and relatives	Malnutrition
Wu et al. [42] (2022)	Singapore	Multicomponent: Community-based e-Health Program consisting of health education, monitoring, and an advisory system for older adults to manage their chronic conditions. It is an 8-wk intensive program, including face-to-face and eHealth (Care4Senior App) sessions	Self-management of chronic diseases
Zacharia et al. [47] (2020)	Australia	Multicomponent: AusMed Diet Program consisting of 2-wk meal plan with recipes, shopping lists, education and behavior change support materials.	Mediterranean diet pattern for older Australians

### Consumer characteristics

Most studies engaged with older adults [32,35–37,39,41,49,56,61–66], or older adults as well as their caregivers [31,33,34,38,56,52,53] or relatives [48,55]. Only 1 study engaged with just the caregivers in developing [60] and evaluating [40] their educational nutrition information. With regards to the ethnicity of the consumers engaged, only 4 of the studies [36,38,42,44,56] reported their ethnicity, with 3 of the 4 studies engaging a majority of consumers of White or Caucasian descent [36,38,44,56]. The study by Wu et al. [42] was conducted in Singapore (where the main ethnic group is Chinese [67]). In this study, all but 1 of the consumers were of Chinese descent, with this consumer being of Malay descent [42]. The number of consumers engaged in each of these studies differed greatly depending on the phase of the educational nutrition intervention consumers were engaged in, as well as the level of consumer engagement, from 3 consumers [35] to 1598 consumers [43]. Table 3 [[31], [32], [33], [34], [35], [36], [37], [38], [39], [40], [41], [42], [43], [44], [45], [46], [47], [48], [49], [50], [51], [52], [53], [54], [55], [56], [57], [58], [59], [60], [61], [62], [63], [64], [65], [66]] describes the type of consumers involved across the studies, including their age, ethnicity, and gender demographics.

**TABLE 3:** Consumer characteristics.

Original study/substudies	Type of consumer (s) engaged	No. of consumers/target audience	Age of consumers/target audience.	Gender distribution of consumers/target audience	Ethnicity of consumers
Al-Sultani et al. [39] (2023)	Edentulous patients wearing complete dentures	Phase 1 (compiling the evidence base through focus groups and literature review): *N* = 10; phase 2–5: no consumers/target audience involved; phase 6 (face validity of prototype leaflet): *N* = 6	Phase 1: aged 52–85 y (mean age = 69.4 y, no SD reported); phase 2–5: no consumers/target audience involved; phase 6: aged between 57 and 84 y (mean age = 73.3 y, no SD reported).	Phase 1: 4 females (40%) and 6 males (60%); phase 2–5: no consumers/target audience involved; phase 6: 4 females (66.7%) and 2 males (33.3%)	Not reported
Chaboyer et al. [43,50,51] (2016)	Phase 1 (Pressure Ulcer Prevention Care Bundle (PUPCB) development): consumers (no details provided on whether they were patients or caregivers or if they were at risk of pressure injury themselves); phase 2 (piloting of PUPCB and qualitative interviews): patients, pressure ulcer risk status was not a criterion; phase 3 (cluster randomized trial): patients at risk of pressure ulcer; phase 4 (process evaluation): patients on study wards at intervention sites, but some data (such as recruitment data) were also collected on patients at control sites.	Phase 1: *N* = 7; phase 2 (piloting of PUPCB): *N* = 58 and (qualitative interviews): *N* = 11; phase 3: intervention - *N* = 799; control - *N* = 799; phase 4: *N* = 19	Phase 1: not reported; phase 2: median age = 71.5 y (IQR: 31.0 y); subsample of participants who participated in the qualitative interviews were 2.8 y older; phase 3: intervention group - median age = 70.0 y (IQR: 20.0 y), age range 18–100 y; control group – median age = 74.0 y (IQR: 22.0 y), age range 19–104 y; phase 4: not reported.	Phase 1: not reported; phase 2: specific numbers not reported, noted more than half of the participants in the larger group (of *N* = 58) were female; phase 3: intervention - 393 females (49.2%) and 406 males (50.8%); control - 434 females (54.3%) and 365 males (45.7%); phase 4: not reported	Not reported
Chudyk et al. [36] (2021)	Patients (who underwent the cardiac surgery procedure within the previous 2 y (2017–2018) at the study hospital and consented to be listed in a database of individuals interested in participating in future research) and their caregivers.	*N* = 10 (6 patients and 4 caregivers -> each caregiver was a patient's spouse)	Median age of patients (*N* = 6): 74 y (IQR: 72–76 y); caregivers’ age (*N* = 4) were not reported.	Gender distribution of the patients was reported (*N* = 6): 3 females (50%) and 3 males (50%); caregivers’ gender distribution (*N* = 4) was not reported.	Patients: 83% (5/6) 83% (5/6) were of White/Caucasian/European descent, and 1 was of First Nations/Inuit/Metis descent caregivers: 100% (4/4) were of White/Caucasian/European descent.
Coales et al. [35] (2023)	Expert patients living with type 2 diabetes who answered “yes” to either of the first 2 questions of the Binge-Eating Disorder-7 Scale confirming experience of episodes of excessive overeating in the past 6 mo and associated distress.	*N* = 3	Expert patients were all aged 65 y and above, no specific age/age range reported.	Not reported	Not reported
Donald et al. [31,33,34,38,52,53,54] (2021)	Phase 1a (scoping review): Can-SOLVE CKD (Canadians Seeking Solutions and Innovations to Overcome Chronic Kidney Disease) patient partners with chronic kidney disease (CKD) and caregivers; Phase 1b (national survey): Can-SOLVE CKD patient partners; phase 2a (focus groups and telephone interviews): patients with CKD and their caregivers; phase 2b (consensus workshop): patients with CKD and their caregivers, Can-SOLVE CKD patient partners; phase 3a (environmental scan): no specific consumers involved but it was noted that Can-SOLVE CKD Network Patient Partners were listed as authors for this study; phase 3b (codesign and usability testing, comprising of steps 1–3)—Step 1 -> creation of website features and content: patients with CKD and their caregivers; Step 2 -> heuristic testing: no consumers/target audience involved; Step 3 -> usability testing: patients with CKD and their caregivers	Phase 1a: not reported; phase 1b: not reported; phase 2a: *N* = 48 (33 patients, 15 caregivers); phase 2b: *N* = 24 (11 patients, 6 caregivers, 2 nurses, 1 dietitian, 1 pharmacist, 1 policymaker, 1 primary care physician and 1 nephrologist), 6 Can-SOLVE CKD patient partners (1 is a caregiver, and 5 are patients with CKD); phase 3a: no specific consumers involved but it was noted that 6 Can-SOLVE CKD Network Patient Partners were listed as authors for this study; phase 3b - step 1: *N* = 18 (14 patients, 4 caregivers); step 2: no consumers/target audience involved; step 3: *N* = 5 (4 patients, 1 caregiver)	Phase 1a: not reported; phase 1b: not reported; phase 2a: patients – under 50 y: 10 patients (30%); 50–64 y: 8 patients (24%); 65–74 y: 8 patients (24%); ≥ 75 y: 7 patients (22%); caregivers - <50 y: 0 caregivers; 50–64 y: 8 caregivers (53%); 65–74 y: 4 caregivers (27%); ≥ 75 y: 3 caregivers (20%); phase 2b: under 50 y: 11 participants (46%), 50–64 y: 9 participants (38%), 65–74 y: 3 participants (12%) and ≥75 y: 1 participant (4%); no age reported for the 6 Can-SOLVE CKD patient partners; phase 3a: no specific consumers involved but it was noted that 6 Can-SOLVE CKD Network Patient Partners were listed as authors for this study, no age reported; phase 3b - step 1: under 50 y: 2 participants (11%); 50–64 y: 4 participants (22%); 65–74 y: 5 participants (28%) and ≥ 75 y: 7 participants (39%); step 2: no consumers/target audience involved; step 3: under 50 y: 2 participants (40%); 50–64 y: 0 participants; 65–74 y: 3 participants (60%) and ≥ 75 y: 0 participants.	Phase 1a: not reported; phase 1b: not reported; phase 2a: patients – 20 females (60%) and 13 males (40%); caregivers - 10 females (67%) and 5 males (33%); phase 2b: 19 females (79%) and 5 males (21%); no gender distribution reported for the 6 Can-SOLVE CKD patient partners; phase 3a: no specific consumers involved but it was noted that 6 Can-SOLVE CKD Network Patient Partners were listed as authors for this study, no gender distribution reported; phase 3b - step 1: 5 females (28%) and 13 males (72%); step 2: no consumers/target audience involved; step 3: 2 females (40%) and 3 males (60%)	Not reported for all phases except phase 3b: all patients and caregivers were of White descent
Happe et al. [41,55] (2022)	Phase 1 (focus group interviews): geriatric rehabilitation patients and their relatives; phase 2 (prototype evaluation): patients in rehabilitation, from geriatric and cardiology wards	Phase 1: *N* = 17 (15 patients and 2 relatives); phase 2: *N* = 49	Phase 1: patients and relatives – under 70 y: 1 participant (5.8%), 70–74 y: 2 participants (11.8%), 75–79 y: 2 participants (11.8%), 80–84 y: 9 participants (52.9%); 85–89 y: 2 participants (11.8%), 90–94 y: 0 participants, 95–99 y: 1 participant (5.8%); phase 2: mean age 77.8 y (SD 6.2 y), age range 66–94 y.	Phase 1: 10 females (58.8%) and 7 males (41.2%); phase 2: 24 females (49%), 25 males (51%)	Not reported
Ilie et al. [44,56,57] (2023)	Phase 1 (conference): patients, survivors, caregivers; Phase 2 (pilot study): men with a history of non-metastatic prostate cancer; phase 3 (crossover randomized clinical trial): men aged 50–82 y scheduled for curative prostate cancer surgery or radiotherapy (± hormone treatment); Online QoL survey: men with a history of localized prostate cancer in the Maritime provinces in Canada.	Phase 1: actual numbers of patients, survivors and caregivers who attended the conference were not reported; phase 2: *N* = 30; phase 3: *N* = 128 (intervention: *N* = 66; waitlist control: *N* = 62); online QoL survey: *N* = 362	Phase 1: not reported; phase 2: mean age = 68.93 y, age range 56–83 y, no SD reported; phase 3: intervention - median age = 66 y (IQR 60–70 y); waitlist control - median age = 68 y (IQR 61–72 y); online QoL survey: mean age = 68.55 (SD 7.117 y, age range 47–88 y).	Phase 1: not reported; phase 2: 100% males; phase 3: 100% males; online QoL survey: 100% males	Phase 1: not reported phase 2: phase 2: 93.4% (28/30) of the consumers were of White or Caucasian descent phase 3: 91% (60/66) of consumers in the PC-PEP intervention were of white descent, whereas 98% (61/62) of consumers in the waitlist control group were of white descent online QoL survey: not reported
Jongstra et al. [37,58,59] (2017)	Phase 1 (conceptual framework): no consumers/target audience involved; phase 2 (platform concept and functional design): older people with elevated cardiovascular disease risk and basic computer skills; phase 3 (platform building—software and content): no consumers/target audience involved; phase 4—testing, pilot study and evaluation: testing—Dutch older people representative for the target population; Pilot/evaluation—participants were aged ≥65 y and had an elevated risk for cardiovascular disease and basic computer skills.	Phase 1: no consumers/target audience involved; phase 2: *N* = 40; phase 3: No consumers/target audience involved; phase 4: testing—not reported; pilot – *N* = 41 (intervention: 29; control: 12); evaluation – *N* = 27	Phase 1: no consumers/target audience involved; phase 2: not reported; phase 3: no consumers/target audience involved; phase 4: testing - not reported; pilot – mean age = 69 y (SD 4.6 y), no age range reported; evaluation – not reported.	Phase 1: no consumers/target audience involved; phase 2: not reported; phase 3: no consumers/target audience involved; phase 4: testing - not reported; pilot – 23 females (56%) and 18 males (44%); evaluation – not reported	Not reported
Papachristou et al. [40,60] (2018)	Informal caregivers looking after people with dementia at home and who manage their food-related processes.	Phase 1 (development of booklet): *N* = 20; phase 2 – (evaluation of booklets): *N* = 20	Phase 1: not reported; phase 2 – (evaluation of booklets): mean age = 70 y, no SD or age range reported.	Phase 1: 10 females (50%) and 10 males (50%); phase 2 – (evaluation of booklets): 10 females (50%) and 10 males (50%)	Not reported
Payne et al. [46,[61], [62], [63]] (2021)	Phase 1 (prototype intervention) and phase 2 (“Think Aloud” and process evaluation interviews): free-living adults aged ≥65 y, with 1 or more health or social conditions associated with malnutrition risk; phase 3 (feasibility study): adults aged ≥65 y who have 1 more major medical or social problems known to increase nutritional risk; phase 4 (effectiveness study): patients aged ≥75 y who are either living alone or have 1 or more major medical or social problem (s) known to increase nutritional risk	Phase 1: *N* = 23 phase 2: “Think Aloud” Interviews – *N* = 23; process evaluation interviews – *N* = 18; phase 3: no published data yet; planned sample size = 150; phase 4: ongoing trial, planned sample size = 7400	Phase 1: 65–74 y: 4 participants (17%); 75–84 y: 12 participants (52%); 85–94 y: 6 participants (26%); missing data: 1 participant (4%); phase 2: “Think Aloud” Interviews - 65–74 y: 4 participants (17%); 75–84 y: 12 participants (52%); 85–94 y: 6 participants (26%); missing data: 1 participant (4%); process evaluation interviews – 65–74 y: 8 participants (44%), 75–84 y: 9 participants (50%), 85–94 y: 1 participant (4%), missing data: 0 (0%); phase 3: no published data yet; phase 4: ongoing trial, no published data yet.	Phase 1: 16 females (69%) and 7 males (30%); phase 2: “Think Aloud” interviews: 16 females (69%) and 7 males (30%); process evaluation interviews – 11 females (61%) and 7 males (39%); phase 3: no published data yet; phase 4: ongoing trial, no published data yet	Not reported
Price et al. [32,49] (2009)	Phase 1 (focus groups): adults from a database compiled by the Oxford Centre for Diabetes Endocrinology and Metabolism clinical research unit of individuals expressing interest in taking part in research studies.; phase 2 (pilot RCT): individuals at increased cardiovascular disease risk, but not known to have cardiovascular disease.	Phase 1: *N* = 21 phase 2: *N* = 194 (intervention: *N* = 99; control: *N* = 95)	phase 1: mean age = 66 y (range = 47–76 y; no SD reported) phase 2: median age = 62.3 y (IQR 54.9–66.1 y).	Phase 1: 8 females (38%) and 13 males (62%) phase 2: 64 females (33%) and 130 males (67%)	Not reported
Roberts et al. [45] (2016)	Phase 1 (developing the pilot intervention): lay adult readers; phase 2 (pilot randomized control trial) and phase 3 (patient interviews): patients admitted to either of the 3 medical wards (orthopedic, renal and rehabilitation) at a metropolitan university teaching hospital in Australia.	Phase 1: not reported; phase 2: *N* = 66 (intervention: *N* = 31; control: *N* = 35); phase 3: *N* = 5	Phase 1: not reported; phase 2: intervention - mean ± SD = 69.6 ± 11.6 y; control - mean ± SD = 73.3 ± 14.7 y; no age range reported; phase 3: not reported.	Phase 1: not reported; phase 2: 32 females (48.5%) and 34 males (51.5%); phase 3: not reported	Not reported
Terp et al. [48,[64], [65], [66]] (2022)	Food'n'Go development: older medical patients admitted to the 5 units of the Department of Internal Medicine in a large university hospital; phase 1a and b (needs assessment): patients; phase 2 (logic model of change): consumers/target audience not involved; phase 3 (designing the intervention via individual conversations/workshop with nurses): patients and relatives; phase 4 (production of program components): patients and relatives	Food'n'Go development: *N* = 25 (intervention: *N* = 9; control: *N* = 16); phase 1a: *N* = 25; phase 1b: *N* = 20; phase 2: consumers/target audience not involved; phase 3: individual conversations – *N* = 5 (3 patients, 2 relatives); workshop with nurses – *N* =4; phase 4: *N* = 3	Food'n'Go development: intervention - mean age = 79.4 y; control - mean age = 80.3 y, no SD/age range reported; phase 1a: median age = 81.0 y (IQR 71.5–88.0 y); phase 1b: mean age = 76 y (SD: 14.2 y), no age range reported; phase 2: consumers/target audience not involved; phase 3: individual conversations – mean age of patients = 80 y (no SD/age range reported); age of relatives not reported; workshop with nurses – not reported; phase 4: mean age = 82 y (no SD/age range reported)	Food'n'Go development: intervention - 7 females (77.8%) females and 2 males (22.2%); control – 8 females (50%) and 8 males (50%); phase 1a: 13 females (52%), 12 males (48%); phase 1b: not reported; phase 2: consumers/target audience not involved; phase 3: individual conversations – not reported; workshop with nurses – not reported; phase 4: not reported	Not reported
Wu et al. [42] (2022)	Older adults aged ≥55 y with chronic diseases	Phase 1 (focus groups): awaiting data to be published; phase 2 (design and development): consumers/target audience were not involved; phase 3 (formative, user-centric evaluation): not reported; pilot testing of study intervention: *N* = 12 (intervention: *N* = 8; control: *N* = 4)	Phase 1: awaiting data to be published; phase 2: consumers/target audience were not involved; phase 3: not reported; pilot testing of study intervention: intervention - mean age = 74.4 y (SD 6.22 y); control - mean age = 69.75 y (SD 8.34 y).	Phase 1: awaiting data to be published; phase 2: consumers/target audience were not involved; phase 3: not reported; pilot testing of study intervention: 9 females (75%) and 3 males (25%)	Phase 3: not reported pilot testing of study intervention – intervention group: 100% (8/8) Chinese, 0% Malay control group: 75% (3/4) Chinese, 25% (1/4) Malay
Zacharia et al. [47] (2020)	Older adults aged ≥55 y	Phase 1 (process evaluation consisting of quantitative survey and telephone interviews): quantitative survey – *N* = 17; telephone interviews – *N* = 6; phase 2 (feasibility trial): *N* = 15	Phase 1 (overall process evaluation) – mean ± SD = 71.2 ± 4.2 y; phase 2: mean ± SD = 70.4 ± 6.1 y.	Phase 1 (overall process evaluation): 12 females (70.6%) and 5 males (29.4%); phase 2: 12 females (80%) and 3 males (20%)	Not reported

Abbreviations: QoL, quality of life; RCT, randomized controlled trial.

### Consumer recruitment, reimbursement, and remuneration

Consumers across the studies were recruited through various avenues such as from clinical settings like hospitals [41,43,45,48], rehabilitation centers [55], and clinics [40,44]. Some consumers were recruited from specific databases [36,46,49], or from advertisements made across multiple social media platforms [35,38,47] or in the community [40], or through specific research networks [38] and support groups [35]. Although some consumers were recruited from another related study [39], 1 study recruited consumers from senior activity centers within a specific neighborhood region [42]. In addition, 1 study recruited consumers from across 3 different countries via multiple avenues such as the population registry, registration lists of individuals listed in primary care practices, various clinical settings and through advertisements in the local media, patient organizations and their websites and healthcare centers, as well as in the community [68]. Most studies [[37], [38], [39], [40], [41],[43], [44], [45], [46], [47], [48]] (*n* = 11) did not report if the consumers involved were reimbursed or provided remuneration in any way. Although only 1 study described some form of reimbursement, such as reimbursement for traveling expenses and the provision of drinks and light refreshments [49], among the other 3 studies which reported remunerating consumers for their participation [35,36,42], only 1 disclosed the specific remuneration amount [36].

### Level of consumer engagement

The level of consumer engagement varied across the different studies, with most engaging consumers in >1 phase in the design and/or development of the educational nutrition intervention. Only 1 study [35] engaged consumers within a single phase, although this could be partly attributed to the level of consumer engagement and the type of educational nutrition intervention in which these consumers were engaged. In this study, input from 3 expert patients was sought via online collaboration workshops to adapt an existing resource to a new target audience [35]. Nonetheless, this study did report the engagement of a consumer group to inform the recruitment materials used in their study, although no further details about the consumer group were reported in the article, such as the number and type of consumers that were part of this group, how they were initially recruited, and how they reviewed the recruitment materials and provided their input.

All of the other studies engaged consumers at multiple phases [[36], [37], [38], [39], [40], [41], [42], [43], [44], [45], [46], [47], [48], [49]], with just over half engaging consumers right from the initial development phase in some form of a needs assessment to better understand the needs of their target audience [33,34,36,37,39,42,46,48,65,66,55,57]. All needs assessments used a qualitative method (e.g. focus groups were most commonly used [36,37,39,42,55], followed by semistructured interviews [63]), except in 1 study, which employed a quantitative approach via a comprehensive online survey [57]. Although 1 study used both focus groups and semistructured telephone interviews in their needs assessment phase [33,34], another used a mixed-methods approach [65,66] (i.e. qualitative and quantitative data) in addition to an observational study [48].

Five of the 15 studies directly engaged consumers in the design of educational nutrition information [[36], [37], [38],^48^,^50^], whereas the other studies relied on consumer input obtained from a prior needs assessment [35,39,40,42,46,49,55]. One study engaged lay adult readers to read the educational intervention brochure and provide feedback; however, details about who these lay adult readers were, how they provided their feedback, and what level of feedback was provided were not described [45]. One study sought input from consumers through a conference that was attended by patients, prostate cancer survivors, caregivers, and health care professionals [56]. Discussions from this conference helped inform the resulting educational nutrition program [56], although details of the level of discussions and input from these consumers were unclear. Only 1 study did not seek any direct or indirect consumer input in the development of the educational nutrition intervention, but instead sought their feedback through evaluating the proposed program and educational materials, as well as testing the feasibility of their program through a 2-week feasibility trial [47].

**FIGURE 2:**
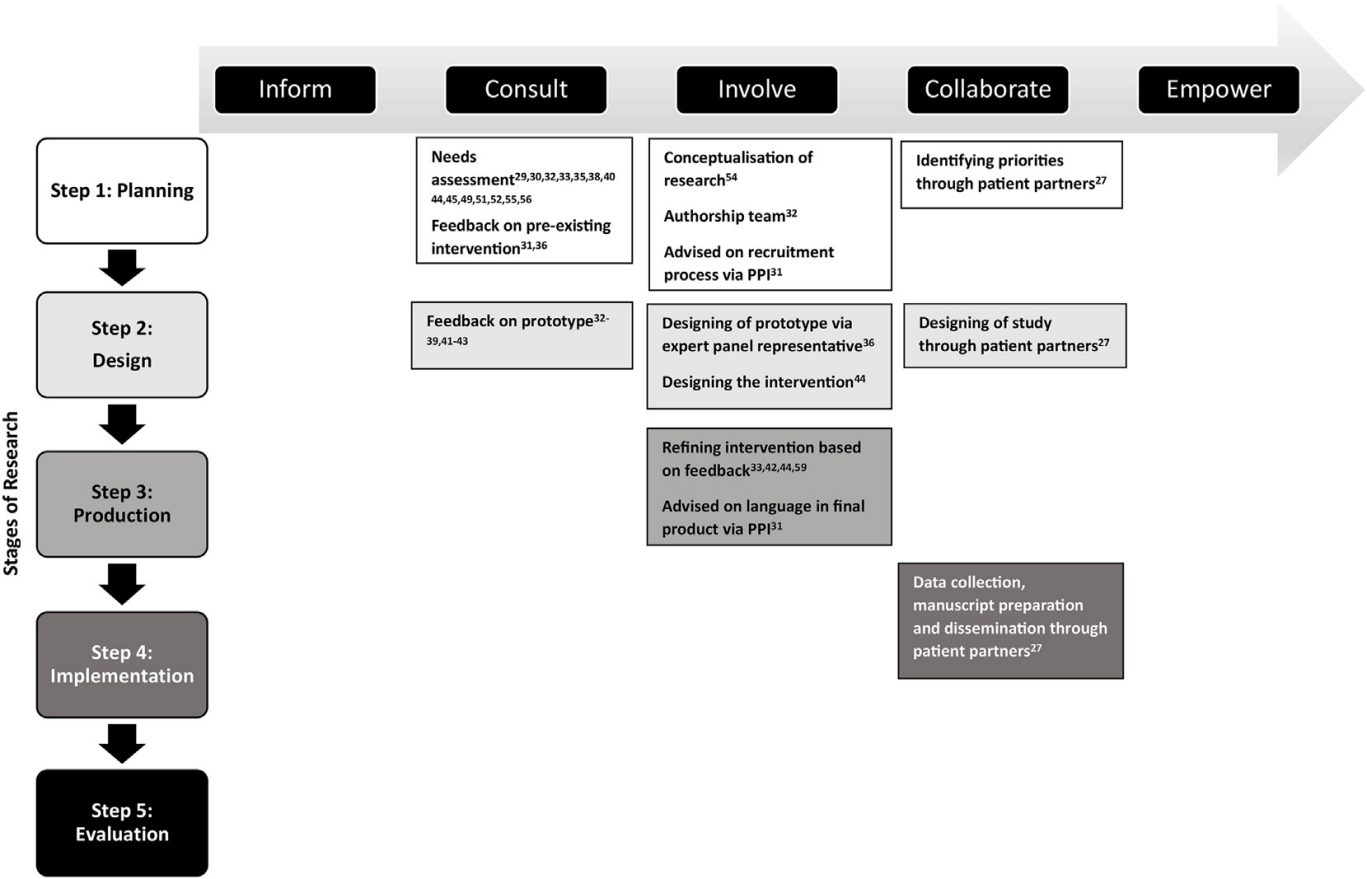
Consumer engagement per IAP2 Public Participation Spectrum at each research stage. PPI, public and patient involvement.

Across all 15 studies, consumer participation was at the "Consult" level through consumer input via a needs assessment and/or feedback on the intervention or prototype proposed in some of the studies. Eight of these 15 studies also engaged consumers on the “Involve” level of participation as consumers were involved in decision making to some degree, either through designing [40,48] or refining the intervention or prototype [37,46,50], conceptualization of research [38], providing input in the study’s recruitment process and language used in the final end-product [35], or engagement as part of the authorship team [36]. Only 1 study reached the "Collaborate" level of consumer engagement through collaborating with patient partners in identifying priorities, designing of study, data collection, and manuscript preparation and dissemination [38]. A summary of the level of consumer engagement across the studies based on the IAP2 Public Participation Spectrum is shown in Figure 2.

Of the 15 studies, it is worth highlighting that only 1 study included consumers as partners in all phases of their study in the form of patient partners [38]. Six patient partners were recruited as collaborators on the CKD self-management research team, of which 5 were patients with CKD and 1 was a caregiver [31]. In addition to providing input into a scoping review [53] and reviewing questions and results obtained in a self-administered, semistructured electronic survey to identify and collate self-management resources used by CKD clinics across Canada for adults with CKD [52], these patient partners were also engaged in the design, data collection, review of results and manuscript preparation and dissemination of a consensus workshop employed to explore preferences for content and features for an e-health tool for CKD self-management support [31,38]. Two of these 6 patient partners also provided input to an interview guide in a qualitative substudy to understand behaviors of patients with CKD and their caregivers and identify potential intervention approaches to support CKD self-management [34]. Furthermore, these 2 patient partners also contributed to the review of the themes obtained during the data analysis of another qualitative substudy which aimed to identify and describe the needs of patients with CKD and informal caregivers for CKD self-management support [33].

### Impact of consumer involvement

Fourteen of the 15 studies obtained input from consumers to inform the design of the educational nutrition information or prototype [[35], [36], [37], [38], [39], [40], [41], [42], [43],[45], [46], [47], [48], [49]]. Of these, 13 studies reported how consumer input helped inform the design of educational nutrition information in terms of the content [[35], [36], [37], [38], [39], [40], [41], [42], [43],[46], [47], [48], [49]], design [[36], [37], [38],^41^,^47^,^49^], wording [35,43,48], and platform [35,37] of the educational nutrition information presented, whereas 1 study did not mention how and what feedback were specifically sought, and how the feedback was then used to refine their educational brochure [45]. Only in 1 study [44] was consumer feedback and input not formally sought to modify or adapt the proposed educational nutrition intervention. However, consumers did provide input to the initial development of the intervention through attendance at a conference [56], although it was unclear as to the extent and level of consumer input provided.

### Evaluation of educational nutrition intervention or prototype

All but 1 study [35] engaged consumers in either providing feedback and/or evaluating the educational nutrition intervention or prototype in various ways, such as through qualitative interviews [39], a Think-Aloud method [37,38,40,41,46], a pilot trial [32,42,43,45,56,58], a feasibility trial [47,62], usability testing [38,41], and/or an effectiveness trial [43,44,61,59]. Although 1 study evaluated the acceptability, feasibility, and preliminary efficacy of 1 of the components (i.e. the Food’n’Go tablet-based application) of their educative nutrition intervention [64], the authors also mentioned an evaluation of their overall intervention in terms of its feasibility and effectiveness [48]; however, details of this evaluation could not be located. For the study that did not engage consumers for formal feedback or evaluation on their intervention, future plans to pilot their adapted online intervention to test its feasibility and acceptability were mentioned, and if recruitment and outcome parameters of this pilot are promising, the authors also have plans to conduct a full-scale trial to assess the effectiveness of their intervention [35].

### Evaluation of consumer engagement

Only 2 studies conducted some form of evaluation with consumers regarding their experience of being engaged as a consumer [31,46]. For instance, face-to-face process evaluation interviews were conducted with participants to evaluate their experience in a feasibility study [46,62]. Participants were asked about their appetite and eating patterns and habits, their views about the intervention materials developed, and their experience in taking part in a feasibility study [68]. Similarly, in the study by Donald et al. [31], participants were evaluated on how satisfied they were with a consensus workshop which was used to identify preferences for content and features for an e-Health tool to support CKD self-management [31]. A workshop satisfaction survey was completed by all participants evaluating the workshop, facilitators, and materials used [69].

## Discussion

### Summary of evidence and implications to practice

This scoping review explored and synthesized how consumers were engaged in the design of different types of educational nutrition information for older adults, as well as mapped out the impact consumer engagement had on the resulting educational information presented. The methods used to engage consumers, as well as the extent and level of consumer engagement varied across the different studies, with a majority of the studies engaging consumers within the “Consult” and “Involve” IAP2 levels. These findings are consistent with the review by Wiles et al. [70], where a consultative approach was adopted in most of the consumer engagement strategies that occurred during the developmental phase of interventions targeted to health services. This common lack of deeper and more extensive consumer engagement could be attributed to various factors such as limited time and resources, and a lack of researcher experience in consumer engagement which can result in tokenistic consumer involvement [71].

In addition, the lack of quantifiable or explicitly reported evidence on the impact, benefits, and effectiveness of consumer engagement on research and health outcomes could be another motivation barrier for studies to engage consumers [71], and future research along with clearer reporting guidelines are needed to bridge this gap. For instance, the systematic review conducted by Fergusson et al. [72] reported a very low prevalence of consumer engagement in health-related interventional studies, with only 23 trials reporting patient engagement activities among the 2777 citations reviewed at full text. It was suggested by the authors that their review was limited by the information reported in publications, and that research teams might have engaged with patients, but failed to report the patient engagement activities in their publication, as their focus was on the results of their studies.

Our review also found that many studies failed to report on how consumers were compensated for their engagement, with only 4 studies describing some form of consumer reimbursement [49] or remuneration [35,36,42]. This is consistent with the findings by Meloncelli et al. [22] where only a third of the studies in their scoping review reported compensation for consumer involvement. Thus, greater recognition should be given to consumers for involvement, to better acknowledge consumers for their time and contributions. Depending on the context of consumer contribution, this should be individualized and could be done through either monetary reimbursement and/or formal acknowledgments such as through authorship teams, to ensure consumers’ input is adequately recognized.

Nonetheless, it was encouraging to discover how most studies engaged consumer throughout multiple phases, with only 1 study involving consumers in just a single phase [35], which was unsurprisingly given that this study aimed to adapt current existing educational information, rather than designing and developing brand new educational information. It is also worth highlighting the impact consumer input had in terms of informing the design of educational nutrition information, with 14 of the 15 studies [[35], [36], [37], [38], [39], [40], [41], [42], [43],[45], [46], [47], [48], [49]] obtaining consumer input to inform the educational nutrition information in terms of its content, wording, design, and/or intervention platform used. This validates consumers’ input to be useful and important rather than tokenistic and that their input was considered in these studies to make an impact on the educational nutrition information presented.

Although most studies engaged consumers in either providing feedback and/or evaluating the educational nutrition intervention or prototype in various ways [[36], [37], [38], [39], [40], [41], [42], [43], [44], [45], [46], [47], [48], [49]], only 2 studies conducted some form of evaluation with consumers regarding their experience in being engaged in the design of the educational nutrition information [31,46]. Overall, little is known about consumers’ experiences as participants across most of the studies, and what could be done to improve their overall experience and engagement. Harrison et al. [73] support this finding, highlighting the lack of a validated tool to evaluate the process of consumer engagement in research, and their experience of being engaged as consumers [73]. Enabling a feedback loop for consumers as participants [74] is important, to provide opportunities to evaluate their experience throughout the engagement process, as well as to determine whether there were any other areas for improvements to allow for deeper and more meaningful consumer engagement. Previous reviews have also highlighted the lack of evaluation on the success or effectiveness of consumer engagement in research studies [21,75,76], suggesting a need to develop more standardized methods to evaluate consumer engagement [75]. This is important to ensure that future consumer engagement activities can be better tailored to suit each particular context across the different studies [75,77]. Furthermore, only 4 of the studies reported on the ethnicity of the consumers engaged [36,38,42,44,56], with a majority of the consumers of White or Caucasian descent, except for the study conducted in Singapore where the main ethnic group was Chinese and thus a majority of the consumers engaged were Chinese [42]. Therefore, greater transparency across all the studies is needed to provide a clearer idea on how well minority ethnic groups are being represented. Additionally, improved engagement with consumers from culturally diverse backgrounds is needed to ensure that their unique needs and preferences are identified and met.

Our consumer advisor also reiterated that clearer reporting on consumers’ actual impact on the research process or intervention would have been insightful, which is a consistent finding among other similar reviews on consumer engagement [21,22,72]. Specific details on how the authors across the studies utilized the consumer input or feedback received would have been useful to know, to better demonstrate the actual impact consumers had on the educational nutrition information presented. Nonetheless, the review by Wiles et al. [70] assessed the effects on engaging consumers in healthcare policy, research and services, and found promising benefits with regards to consumer engagement. There was evidence that engaging consumers in developing and implementing health services to improve the care of mothers and their infants resulted in a reduction in neonatal mortality [70]. In addition, consumer engagement in the development of patient informational resources contributes to material that is more relevant and reader-friendly and can improve knowledge [70]. Consumer engagement was also reported to play a role in recognizing a wider range of healthcare priorities that complement those from healthcare professionals [70].

### Consumer engagement across the studies from a consumer’s perspective

During in-depth discussions with our consumer advisor, the level of engagement of consumers at the “Involve” stage was questionable in some studies from a consumer’s perspective. For instance, in the study by Chudyk et al. [36], although agreement was reached on categorizing this study’s consumer engagement level as “Involve,” the actual level of engagement by consumers who were on the advisory panel as coauthors was unclear. For example, it was not clear which stages of the research study these consumers were involved in, and what their contributions were across the whole study as coauthors. Given the lack of clarity in reporting, there was uncertainty related to the coauthorship status attributed. Likewise, for the studies by Coales et al. [35] and Papachristou et al. [40], our consumer advisor highlighted that details on the actual level of engagement from the consumer group and dementia caregiver representative (as part of an expert panel of 3), respectively, were lacking, in terms of the level of decision-making these consumers had, and what impact their engagement had on the educational nutrition information presented. Nonetheless, our consumer advisor commended the study by Donald et al. [38] which reached the "Collaborate" level of consumer engagement as there was engagement with caregivers of patients with CKD, in addition to patients with CKD. This was regarded positively as caregivers often provide a different perspective to patients from a caregiver’s point of view, which is useful in ensuring that any educational nutrition information presented is holistic and better addresses the needs of the target audience.

### Recommendations for future practice and research

Moving forward, stronger authentic partnerships with consumers could be forged by providing consumers with more decision-making opportunities. As evidenced in this review, most studies had consumer involvement at the "Consult" and "Involve" level, with only 1 study involving consumers at a deeper "Collaborate" level [38]. Further meta-analyses of existing relevant studies to quantify the potential effects of increased consumer engagement across the IAP2 spectrum would be encouraging and pivotal for researchers looking to employ consumer engagement strategies. Enabling increased decision-making capacity for consumers involved in research not only creates enhanced partnerships but also allows for greater ownership of the results. This may lead to improved outcomes because more tailored educational nutrition information that better addresses their specific needs and concerns is produced. Admittedly, this can sometimes be difficult to achieve in practice, as a hierarchy of power exists between health professionals and healthcare consumers, so inadvertently power imbalances result [78], which can impact on the eventual level of influence consumers have. However, by placing greater trust in consumers and by recognizing the value of their unique lived experiences, more equal partnerships with consumers can be built. Putting greater emphasis on shared goals and shared experiences and by ensuring consumers are validated and acknowledged throughout the process of their engagement, a more collaborative and power-sharing environment is created in which consumers’ needs are adequately listened to and addressed.

Our consumer advisor also highlighted that consumer engagement could be improved if researchers engaged consumers right from the start of their research project rather than only at certain time points, which echoes the recommendation by Puts et al. [79]. This would require forward planning and seeking consumer input at the early "ideas" stage when planning the research project, thus allowing consumers to be part of the research team right from the very start. It was also encouraged by our consumer advisor that researchers be selective with the type of consumers they engage for their research, as there is no “one size fits all” approach. Consumers should also be provided with just enough information to understand their role and responsibility in the research team, as too much information, especially at the start, can be overwhelming for consumers, which is consistent with the findings by Todd and Nutbeam [77]. The researcher leading the project should ideally be organized, able to communicate well, and actively promote shared decision-making with the consumers involved, to allow for a trusting and empowering relationship to be built between the research team and the consumers involved. As this is dependent on the experience and expertise of the researcher, proper researcher training and education in consumer engagement is important to facilitate more effective consumer engagement [71,73]. This would allow consumers to freely share their opinions, and exchange ideas and knowledge with researchers in the spirit of colearning, thus promoting a more equal and robust research process. The result may potentially lead to more user-friendly, tailored, nutrition educational interventions. Channeling adequate funding toward consumer engagement can also prevent consumer engagement that is tokenistic [71], because adequate resources are allocated for more meaningful consumer engagement.

In addition, it is worth noting that many factors aside from adequate nutrition knowledge and information can influence an individual’s eating behavior. Behavior change strategies such as environmental cues and behavioral nudges (e.g. placement of healthy foods [80], manipulating the prominence of healthy choices [81], healthy eating reminders [82]) can also influence dietary choices and future research could explore how they can be incorporated into interventions for older adults alongside well-designed nutrition information.

### Strengths and limitations

A key strength of this review is its methodological rigor that is in line with the standards and guidance for scoping review conduct and reporting, as well as the use of 3 independent authors in the article screening process. Furthermore, double extraction was employed to minimize error, with 2 independent authors coming together after extracting all relevant information for this review. A third author provided input if consensus could not be reached on any of the data extracted. Additionally, a comprehensive search strategy was employed, and a wide scope of all available evidence was included to ensure a good overview of the topic being explored. Importantly, this review included perspectives from a consumer advisor, which is invaluable in providing an alternative outlook on how studies engage consumers and the impact of consumer involvement from a consumer’s point of view. The unique contributions of our consumer advisor, in addition to the reflections from our research team and our consumer advisor, are outlined in Table 4.

**TABLE 4:** Consumer advisor’s contributions and research team’s/consumer advisor’s reflections.

Consumer advisor’s role and influence on this review	Research team’s reflections on consumer advisor’s involvement on this review	
	Benefits	Challenges
- Provided suggestions on what specific areas and how she would like to contribute as an active healthcare user and consumer - Provided a consumers’ perspective on: ○ how genuine the consumer input and involvement was across the studies ○ reviewed the IAP2 classifications for each study ○ suggested areas for improvements to enhance consumer engagement in future research ○ suggested additional journal articles relevant to consumer engagement to the research team that would be worthwhile reading to improve our knowledge and skills in consumer engagement - 1 article was subsequently included in the discussion of this article - Reviewed final draft of scoping review to ensure that her input and contributions had been accurately captured and acknowledged throughout this review - Provided her reflections on her involvement in this review in terms of the benefits obtained and the challenges experienced	- Our research team gained an alternative perspective on interpreting the findings of our scoping review from a consumer’s point of view. - There is an enhanced level of confidence when assessing studies on their level of consumer engagement based on the IAP2 Public Participation Spectrum with input from a consumer. - Valuable, practical and honest insights were provided by our consumer advisor on how to improve consumer engagement in future research studies, as well as within our team. - Various colearning opportunities were evident throughout the discussions held with our consumer advisor which improved our research team’s skills and knowledge with regard to consumer engagement. - We have also forged a strong and trusting relationship with our consumer advisor who is keen to work on future related projects.	- Our consumer advisor was initially presented with the original version of our extraction table to provide an overview of the studies included in this review. However, this version was not user-friendly and upon further discussions, refinements were made to the formatting of the table in terms of font size and table layout to improve the readability of the data in this table. In addition, tables used in this review that comprised more concise information from the 15 original studies were shared with our consumer advisor to enhance her understanding of the evidence extracted from the studies in this review. - Our research team did not initially plan to involve a consumer in this review from the very start and only decided on doing so mid-way when analyzing the results of the review. On hindsight, consumer engagement could have been made more meaningful by engaging our consumer advisor right from the beginning, as she may have had alternative views on the search terms for our search strategy and her input as a consumer in approaching this scoping review from the very start would have been useful.
	Consumer advisor’s reflections on her involvement on this review	
	Benefits	Challenges
	• Valued being asked to participate in this project as a consumer. • Found it easy to establish an easygoing, trusting relationship with the PhD candidate within just a few meetings. • Appreciated receiving hard copies of the journal articles to read, which was the preferred option and made the process easier. • Valued flexibility when organizing online meetings to match both our diaries. • Received timely, encouraging feedback and communications about my contributions. • It was helpful to receive table summaries of study characteristics, types of consumers involved, and IAP2 level of involvement, especially given the quick turnaround required. • Learned more about the methods other researchers used to engage with consumers in the various studies I read. • Valued the opportunity to contribute to the review article before submission for publication.	• Could possibly have been engaged earlier in the process. For example, suggestions could have been made on suitable keywords to use in the search strategy for this scoping review, and assistance may have been provided in determining which papers to include. • At the beginning, there could have been more clarity around expectations, time commitment required, and reimbursement processes from the research team. However, these were discussed and subsequently successfully addressed.

Abbreviation: IAP2, International Association for Public Participation.

Thorough reporting on the level and nature of consumer involvement in this review was supported with the use of the GRIPP2 guidelines, although as highlighted by Meloncelli et al. [22], direction on the terminology and definitions could be improved. A limitation of this review is the exclusion of grey literature where consumer engagement may be more prevalent. Furthermore, the level of consumer engagement on this review could also have been improved by engaging with our consumer advisor right from the very start rather than after the search strategy has been set up and the screening process was underway, as she may have had other suggestions on approaching this scoping review such as alternative keywords to be used in our search strategy. Nonetheless, the relationship built between the authors of the team and our consumer advisor has been a positive one, with various opportunities for colearning throughout our team discussions.

### Conclusion

This scoping review has found a low level of consumer engagement in the design of educational nutrition information for older adults and caregivers with little involvement or genuine collaboration seen in most of the studies to date. Although input from consumers has helped to inform the design of the educational nutrition information in terms of content, design, wording, and platform, deeper and more authentic consumer engagement can allow for more tailored educational nutrition resources to be developed. This potentially translates to improved nutrition and better health outcomes. Greater emphasis needs to be placed on shared decision-making with consumers, and the level of consumer engagement should aim to move further toward the “Empower” end of the IAP2 Spectrum. Forward planning to collaborate with consumers right from the start is key to ensuring adequate funding and resources for more meaningful consumer engagement. Improved recognition of consumers’ input and contributions through clearer reporting of the impact of their input is also paramount in acknowledging consumers for their time and effort. Consumers have a wealth of lived experiences and knowledge that should be tapped into to ensure that any educational nutrition information designed for them best addresses their needs and preferences.

## Author contributions

The authors’ contributions were as follows – AL, AMY, MDM: contributed to the conception and design of this review; AL, CH, MELH: screened the articles; AL, CH: extracted the articles; EMM: assessed the consumer engagement component of all included studies and provided insight for future improvements to consumer engagement from a consumer’s perspective; AL: drafted the original article, made revisions and primary responsibility for the final content; AMY, CH, EMM, MELH, MDM: revised drafts and provided critical feedback; and all authors: read and approved the final manuscript.

## Data availability

Data described in the manuscript will be made available on request.

## Funding

Research reported in this publication was supported by the Medical Research Future Fund (MRFF) under grant number MRF2016045.

## Conflict of interest

The authors report no conflicts of interest.
