# Supplementary material for: Consumer Engagement in the Design of Educational Nutrition Information for Older Adults and Their Caregivers: A Scoping Review
Source: Adv Nutr. 2025 Mar 6;16(4):100401. doi: 10.1016/j.advnut.2025.100401 (PMC11986611; doi:10.1016/j.advnut.2025.100401)
Supplement: Multimedia component 1 [file mmc1.pdf]

Supplementary Table 1: Preferred Reporting Items for Systematic reviews and Meta-Analyses extension for Scoping Reviews (PRISMA-ScR) Checklist

SECTION	ITEM	PRISMA-ScR CHECKLIST ITEM	REPORTED ON PAGE #
**TITLE**			
Title	1	Identify the report as a scoping review.	1
**ABSTRACT**			
Structured summary	2	Provide a structured summary that includes (as applicable): background, objectives, eligibility criteria, sources of evidence, charting methods, results, and conclusions that relate to the review questions and objectives.	2 to 3
**INTRODUCTION**			
Rationale	3	Describe the rationale for the review in the context of what is already known. Explain why the review questions/objectives lend themselves to a scoping review approach.	8 to 9
Objectives	4	Provide an explicit statement of the questions and objectives being addressed with reference to their key elements (e.g., population or participants, concepts, and context) or other relevant key elements used to conceptualize the review questions and/or objectives.	8 to 9
**METHODS**			
Protocol and registration	5	Indicate whether a review protocol exists; state if and where it can be accessed (e.g., a Web address); and if available, provide registration information, including the registration number.	9
Eligibility criteria	6	Specify characteristics of the sources of evidence used as eligibility criteria (e.g., years considered, language, and publication status), and provide a rationale.	9 to 11
Information sources*	7	Describe all information sources in the search (e.g., databases with dates of coverage and contact with authors to identify additional sources), as well as the date the most recent search was executed.	11 to 12
Search	8	Present the full electronic search strategy for at least 1 database, including any limits used, such that it could be repeated.	Supplementary File S3
Selection of sources of evidence†	9	State the process for selecting sources of evidence (i.e., screening and eligibility) included in the scoping review.	11 to 12
Data charting process‡	10	Describe the methods of charting data from the included sources of evidence (e.g., calibrated forms or forms that have been tested by the team before their use, and whether data charting was done independently or in duplicate) and any processes for obtaining and confirming data from investigators.	12
Data items	11	List and define all variables for which data were sought and any assumptions and simplifications made.	12
Critical appraisal of individual sources of evidence§	12	If done, provide a rationale for conducting a critical appraisal of included sources of evidence; describe the methods used and how this information was used in any data synthesis (if appropriate).	N/A
Synthesis of results	13	Describe the methods of handling and summarizing the data that were charted.	12

SECTION	ITEM	PRISMA-ScR CHECKLIST ITEM	REPORTED ON PAGE #
**RESULTS**			
Selection of sources of evidence	14	Give numbers of sources of evidence screened, assessed for eligibility, and included in the review, with reasons for exclusions at each stage, ideally using a flow diagram.	13 to 14
Characteristics of sources of evidence	15	For each source of evidence, present characteristics for which data were charted and provide the citations.	14 to 20
Critical appraisal within sources of evidence	16	If done, present data on critical appraisal of included sources of evidence (see item 12).	N/A
Results of individual sources of evidence	17	For each included source of evidence, present the relevant data that were charted that relate to the review questions and objectives.	14 to 20
Synthesis of results	18	Summarize and/or present the charting results as they relate to the review questions and objectives.	14 to 20
**DISCUSSION**			
Summary of evidence	19	Summarize the main results (including an overview of concepts, themes, and types of evidence available), link to the review questions and objectives, and consider the relevance to key groups.	20 to 23
Limitations	20	Discuss the limitations of the scoping review process.	26 to 27
Conclusions	21	Provide a general interpretation of the results with respect to the review questions and objectives, as well as potential implications and/or next steps.	27
**FUNDING**			
Funding	22	Describe sources of funding for the included sources of evidence, as well as sources of funding for the scoping review. Describe the role of the funders of the scoping review.	28

JBI = Joanna Briggs Institute; PRISMA-ScR = Preferred Reporting Items for Systematic reviews and Meta-Analyses extension for Scoping Reviews.

* Where *sources of evidence* (see second footnote) are compiled from, such as bibliographic databases, social media platforms, and Web sites.

† A more inclusive/heterogeneous term used to account for the different types of evidence or data sources (e.g., quantitative and/or qualitative research, expert opinion, and policy documents) that may be eligible in a scoping review as opposed to only studies. This is not to be confused with *information sources* (see first footnote).

‡ The frameworks by Arksey and O'Malley (6) and Levac and colleagues (7) and the JBI guidance (4, 5) refer to the process of data extraction in a scoping review as data charting.

§ The process of systematically examining research evidence to assess its validity, results, and relevance before using it to inform a decision. This term is used for items 12 and 19 instead of "risk of bias" (which is more applicable to systematic reviews of interventions) to include and acknowledge the various sources of evidence that may be used in a scoping review (e.g., quantitative and/or qualitative research, expert opinion, and policy document).

From: Tricco AC, Lillie E, Zarin W, O'Brien KK, Colquhoun H, Levac D, et al. PRISMA Extension for Scoping Reviews (PRISMAScR): Checklist and Explanation. Ann Intern Med. 2018;169:467–473. doi: 10.7326/M18-0850.

Supplementary Table 2. Open Science Framework (OSF) Registry Protocol Deviations/Updates

Date	Changes/updates made	Rationale
18th August 2023	Initial registration of scoping review protocol.	N/A
13th September 2023	Withdrew original OSF registration and re-submitted new registration with updated title and terminologies.<br>- Title was updated from “Consumer involvement in the design of educational nutrition information for older patients and clients” to “Consumer engagement in the design of educational nutrition information for older patients and clients: A scoping review.”<br>- The terms “involve” or “involvement” were replaced with “engage” or “engagement” throughout the protocol.	To minimize confusion with terms used in the International Association of Public Participation (IAP2) Spectrum.
27th September 2023	Updated OSF registration ‘data collection procedures’ section.<br>- Inclusion and exclusion criteria were refined.<br><br>Title of our scoping review have been updated but not amended in current registered OSF protocol as that would require another withdrawal of our registered OSF protocol.<br>- Title was updated from “Consumer engagement in the design of educational nutrition information for older patients and clients: A scoping review.” to “Consumer engagement in the design of educational nutrition information for older adults and their caregivers: A scoping review.”	To provide better clarity and transparency in the article screening and selection process.<br><br>To provide a more inclusive title that more accurately reflects the target population of our scoping review.

Supplementary Table 3. Search Strategy.

Medline via OVID

Search	Query
#1	Patients/ or Caregivers/
#2	(consumer* or patient* or client* or carer* or caregiver* or care-giver* or stakeholder* or "end user*" or "end-user*").ab,kf,ti.
#3	1 or 2
#4	Information Sources/ or Information Technology/ or Mobile Applications/ or Audiovisual Aids/ or Books/ or Books, Illustrated/ or Pamphlets/ or Consumer Health Information/
#5	(material* or handout* or brochure* or website* or book* or apps or app or applications or "mobile app*" or pamphlet* or printout* or "audiovisual aid*" or "audio visual aid*" or "audio-visual aid*" or video* or podcast* or blog* or "information technolog*" or "information source*" or "information resource*").ab,kf,ti.
#6	4 or 5
#7	Nutrition Therapy/
#8	Food/ or Cooking/
#9	Eating/ or Feeding Behavior/
#10	"Diet, Food, and Nutrition"/ or Diet/
#11	(nutrition* or diet* or food* or meal* or nutrient* or cook* or eat*).ab,kf,ti.
#12	7 or 8 or 9 or 10 or 11
#13	Community-Based Participatory Research/ or Community Participation/ or Patient Participation/ or User-Centered Design/ or Patient-Centered Care/
#14	("person-centred" or "person-centered" or "patient-centred" or "patient-centered" or "lived experience*" or "user-centered" or "user-centred" or "collaborative care" or "participat* research").ab,kf,ti.
#15	((consumer* or patient* or client* or carer* or caregiver* or care-giver* or stakeholder* or "end user*" or "end-user*") adj3 (participat* or engag* or involv* or collab*)).ab,kf,ti.
#16	("co-design*" or codesign* or "co-create*" or cocreate* or "co-invent*" or coinvent*).ab,kf,ti.
#17	13 or 14 or 15 or 16
#18	3 and 6 and 12 and 17

Scopus

Search	Query
#1	(consumer* OR patient* OR client* OR carer* OR caregiver* OR care-giver* OR stakeholder* OR "end user*" OR "end-user*")
#2	(material* OR handout* OR brochure* OR website* OR book* OR apps OR app OR applications OR "mobile app*" OR pamphlet* OR printout* OR "audiovisual aid*" OR "audio visual aid*" OR "audio-visual aid*" OR video* OR podcast* OR blog* OR "information technolog*" OR "information source*" OR "information resource*")
#3	(nutrition* OR diet* OR food* OR meal* OR nutrient* OR cook* OR eat*)
#4	("person-centred" OR "person-centered" OR "patient-centred" OR "patient-centered" OR "lived experience*" OR "user-centered" OR "user-centred" OR

	"collaborative care" OR "participat* research") OR ((consumer* or patient* or client* or carer* or caregiver* or care-giver* or stakeholder* or "end user*" or "end-user*") W/2 (participat* or engag* or involv* or collab*)) OR ("co-design*" OR codesign* OR "co-create*" OR cocreate* OR "co-invent*" OR coinvent*)
#5	1 OR 2 OR 3 OR 4

Web of Science

Search	Query
#1	(consumer* OR patient* OR client* OR carer* OR caregiver* OR care-giver* OR stakeholder* OR "end user*" OR "end-user*")
#2	(material* OR handout* OR brochure* OR website* OR book* OR apps OR app OR applications OR "mobile app*" OR pamphlet* OR printout* OR "audiovisual aid*" OR "audio visual aid*" OR "audio-visual aid*" OR video* OR podcast* OR blog* OR "information technolog*" OR "information source*" OR "information resource*")
#3	(nutrition* OR diet* OR food* OR meal* OR nutrient* OR cook* OR eat*)
#4	("person-centred" OR "person-centered" OR "patient-centred" OR "patient-centered" OR "lived experience*" OR "user-centered" OR "user-centred" OR "collaborative care" OR "participat* research") OR ((consumer* or patient* or client* or carer* or caregiver* or care-giver* or stakeholder* or "end user*" or "end-user*") NEAR/3 (participat* or engag* or involv* or collab*)) OR ("co-design*" OR codesign* OR "co-create*" OR cocreate* OR "co-invent*" OR coinvent*)
#5	1 OR 2 OR 3 OR 4

CINAHL

Search	Query
#1	MH Patients OR MH Caregivers
#2	(consumer* OR patient* OR client* OR carer* OR caregiver* OR care-giver* OR stakeholder* OR "end user*" OR "end-user*")
#3	1 OR 2
#4	MH Information Technology OR MH Mobile Applications OR MH Audiovisuals OR MH Books OR MH Electronic Books OR MH Information Resources OR MH Pamphlets
#5	(material* OR handout* OR brochure* OR website* OR book* OR apps OR app OR applications OR "mobile app*" OR pamphlet* OR printout* OR "audiovisual aid*" OR "audio visual aid*" OR "audio-visual aid*" OR video* OR podcast* OR blog* OR "information technolog*" OR "information source*" OR "information resource*")
#6	4 OR 5
#7	MH Nutrition OR MH Nutrition Services OR MH Diet
#8	MH Cooking OR MH Meal Preparation
#9	MH Food or MH Eating
#10	MH Eating Behaviour
#11	MH Food Habits OR MH Diet Therapy
#12	(nutrition* OR diet* OR food* OR meal* OR nutrient* OR cook* OR eat*)

#13	7 OR 8 OR 9 OR 10 OR 11 OR 12
#14	MH Action Research
#15	MH Community Role
#16	MH Consumer Participation
#17	("person-centred" OR "person-centered" OR "patient-centred" OR "patient-centered" OR "lived experience*" OR "user-centered" OR "user-centred" OR "collaborative care" OR "participat* research")
#18	((consumer* or patient* or client* or carer* or caregiver* or care-giver* or stakeholder* or "end user*" or "end-user*") N2 (participat* or engag* or involv* or collab*))
#19	("co-design*" OR codesign* OR "co-create*" OR cocreate* OR "co-invent*" OR coinvent*)
#20	14 OR 15 OR 16 OR 17 OR 18 OR 19
#21	3 AND 6 AND 13 AND 20

PyscINFO

Search	Query
#1	exp Patients/
#2	exp Caregivers/
#3	(consumer* or patient* or client* or carer* or caregiver* or care-giver* or stakeholder* or "end user*" or "end-user*").ab,ti.
#4	1 or 2 or 3
#5	exp Information/ or exp Health Information/
#6	exp Health Information Technology/ or exp Digital Information/ or exp "Information and Communication Technology"/
#7	exp Audiovisual Communications Media/
#8	exp Electronic Books/ or exp Books/
#9	exp Reading Materials/
#10	exp Print Media/
#11	exp Mobile Applications/
#12	(material* or handout* or brochure* or website* or book* or apps or app or applications or "mobile app*" or pamphlet* or printout* or "audiovisual aid*" or "audio visual aid*" or "audio-visual aid*" or video* or podcast* or blog* or "information technolog*" or "information source*" or "information resource*").ab,ti.
#13	5 or 6 or 7 or 8 or 9 or 10 or 11 or 12
#14	exp Nutrition/
#15	exp Food/
#16	exp Food Preparation/
#17	exp Eating Behavior/
#18	exp Diets/
#19	(nutrition* or diet* or food* or meal* or nutrient* or cook* or eat*).ab,ti.
#20	14 or 15 or 16 or 17 or 18 or 19
#21	exp Action Research/ or exp Community Involvement/
#22	exp Client Participation/ or exp Participation/
#23	exp Patient Centered Care/ or exp Client Centered Therapy/
#24	exp Collaboration/

#25	("person-centred" or "person-centered" or "patient-centred" or "patient-centered" or "lived experience*" or "user-centered" or "user-centred" or "collaborative care" or "participat* research").ab,ti.
#26	((consumer* or patient* or client* or carer* or caregiver* or care-giver* or stakeholder* or "end user*" or "end-user*") adj3 (participat* or engag* or involv* or collab*)).ab,ti.
#27	("co-design*" or codesign* or "co-create*" or cocreate* or "co-invent*" or coinvent*).ab,ti.
#28	21 or 22 or 23 or 24 or 25 or 26 or 27
#29	4 and 13 and 20 and 28

Supplementary Table 4. Completed Guidance for Reporting Involvement of Patients and the Public Reporting Checklist 2 – Short Form for inclusion of a consumer as a co-author.

**Section and topic**	**Item**	**Page number**
1: Aim	Report the aim of PPI in the study	11 to 12
2: Methods	Provide a clear description of the methods used for PPI in the study	11 to 12
3: Study results	Outcomes—Report the results of PPI in the study, including both positive and negative outcomes	Table 4
4: Discussion and conclusions	Outcomes—Comment on the extent to which PPI influenced the study overall. Describe positive and negative effects	Table 4
5: Reflections/critical perspective	Comment critically on the study, reflecting on the things that went well and those that did not, so others can learn from this experience	Table 4
